# 

**Published:** 1922-04

**Authors:** 


REVIEW

*Established 1886* No. 1849. *Vol. LXXI.*

*New Series.* Vol. 1. No. 7 *April* 1922 Price Sixpence

### CONTENTS.

| | PAGE |
|---|---|
| NOTES AND COMMENTS :— The Economic Value of Health Services—Dental Fees—The Narrowing English Face—Insurance Economies—The Exclusion of Women Students from the London Hospital—The Quarrels of the Anti-Venereal Disease Societies—The Rockefeller Gift—The Hospitals, &c. | 179 |
| THE DECLINE AND FALL (?) OF TUBERCULOSIS.—I. By Dr. Edgar L. Collis, M.A., M.D., M.R.C.P. | 187 |
| THE A.B.C. OF VITAMINS.—II. By Dr. Harold Scurfield, M.D., D.P.H. | 191 |
| THE FUTURE OF THE VOLUNTARY HOSPITALS By the Rt. Hon. The Earl of Onslow | 192 |
| IS THE PANEL DOCTOR DOING HIS JOB ? By R. W. Harris | 194 |
| HEALTH WITH ECONOMY: THE POSITION ON THE SOUTH COAST. II.—FOLKESTONE | 196 |
| THE ORTHOPÆDIC HOSPITAL AT SHEPHERD'S BUSH | 198 |
| REVIEWS | 199 |
| HOSPITAL FINANCE | 201 |
| HOSPITAL NEWS | 203 |
| CORRESPONDENCE | 205 |
| PARLIAMENTARY QUESTIONS AND ANSWERS | 207 |
| OFFICIAL REPORTS, &c. | 208 |

